# Practical Remarks on Blood-Letting

**Published:** 1866-05

**Authors:** D. B. Trimble

**Affiliations:** Chicago, Ill.


ARTICLE XVI.

### PRACTICAL REMARKS ON BLOOD-LETTING.

By D. B. TRIMBLE, M.D., Chicago, Ill.

In continuation of the subject of the application of blood-letting in the treatment of diseases, I propose, next, to take up those affecting the circulatory system.

In this class will be enumerated the diseases of the heart and its membranes; those of the arteries, and veins; and those of the blood: to which this treatment is applicable.

### 1st. DISEASES OF THE HEART.

In the inflammatory affections of the heart, blood-letting, both general and local, is frequently required: and the different phlegmasiæ affecting this organ being very frequently combined, and the treatment that is applicable to one, to some extent, being applicable to all, it may be supposed that they should be considered under one head; but as they do, frequently, occur separately, and often arise from different causes, it is safer to state the treatment of each disease separately.

*Pericarditis.*—On the Etiology of this disease, in a very great degree, depends the propriety or impropriety of bleeding. If an idio-pathic affection (which is very rarely the case), free venesection will generally give prompt relief, and if followed by other proper remedial measures, including, if necessary, leeching, a speedy and perfect cure may be reasonably expected. If of rheumatic origin, prompt and free venesection is also demanded. This may be the more freely resorted to, if the pericarditis occurs simultaneously with the rheumatism, than if appearing after the patient had been previously affected. Venesection is the better borne in the cases arising from this cause, as the patients are generally young and robust. But even when of rheumatic origin, this remedy should not be too freely resorted to, for, as Dr. WALSHE, in his treatise on Diseases of the Heart, says—"Bleeding from the arm cannot be pushed to any notable extent, without the risk of inducing syncope—an occurrence of serious danger when already the heart's vigor is dynamically and statically impaired." "Very copious depletion, particularly in some constitutions, excites the heart greatly;" but yet he says "Moderate venesection shortens the duration of pericarditis, and does so more effectually the earlier it is employed." He recommends the abstraction of eight to twelve ounces in this way, and "if well supported, followed by the abstraction of six or eight ounces more, by cupping or leeching over the heart." Professor FLINT, in his work on the same subject, says that a person in "fair health," affected with pericarditis from either of the above mentioned causes, is a proper subject for blood-letting at the commencement of the disease. The immediate effects of bleeding are, relief to the pain and dyspnœa, and diminished force and less irregularity of the heart's action: the ultimate effects, if successful, the prevention of effusion into, and adhesion of, the pericardium, and restoration to health. But a large number of cases of pericarditis arise from other causes, and generally of a debilitating character, which contra-indicate the use of blood-letting in many cases, and should induce more caution in all. Among these causes may be enumerated prostration from previous disease, intemperance, anæmia, and albunimuria. The last mentioned is a frequent cause of pericarditis, and as it occurs generally in those advanced in life, bleeding should be dispensed with. Where effusion has taken place, it is also useless and improper to bleed. In chronic pericarditis, where a congested condition of the pericardium exists, local depletion by leeching, or cupping the left thoracic region, may be required, but in the majority of cases it is not necessary.

In *Endo-carditis*, bleeding is as necessary as in the preceding disease, and there is less caution necessary in its application, as there is not the danger of debility, or paralysis of the heart, from too much depletion, that there is in pericarditis. In endo-carditis we must not expect to find the severe pain in the left thoracic region that is symptomatic of pericarditis, for in this disease the pain is more obtuse, and frequently absent. On the other hand, the pulse, in the early stage, is even more frequent than in pericarditis, though failing more early. Congestion of the lungs, brain, &c., often occur in the course of the disease, calling for prompt and active treatment to obviate them. The same conditions of the system, and the same causes, as in pericarditis, modify and govern the treatment, in the same way, in endo-carditis.

In *Endo-Pericarditis*, the same treatment is required as in the preceding cases.

*Carditis*, or *Myo-carditis*, as an idio-pathic affection, is a very rare disease. It is almost always associated with inflammation of either its lining or investing membrane, or with both, and its symptoms are very obscure. Its treatment is almost identical. In bleeding, in these cardiac inflammations, we should have a reference, not only to the preservation of life, and restoration to health, but also to the prevention of their sequelæ; as abscesses, softening, induration, and aneurismal dilatation in myo-carditis; chronic valvular disease in endo-carditis; effusion and adhesion in pericarditis; hypertrophy in each, &c.

*Hypertrophy* of the heart, being a chronic disease, produced by other diseases, especially of the heart, bloodvessels, or blood; or by injurious habits of business or indulgence, does not require the free depletion that is necessary in the active cardiac inflammations. It is generally, though not always, associated with dilatation, and where the latter condition predominates, bleeding must be very cautiously, if at all, used. But in simple hypertrophy, or where this is in excess of the dilatation, especially if consequent upon inflammation of the heart, or its membranes, plethora, rheumatism, or chronic valvular disease, then moderate venesection, repeated at intervals of a few weeks, and accompanied or followed by the use of cups or leeches, would greatly aid in the cure of the disease. When the blood is in an anæmic condition, it would be improper to adopt this practice. WALSHE prefers cupping or leeching over the præcordial region, to general bleeding; but FLINT advises general blood-letting, "when there exists over-repletion of the general vascular system, the object being, by lessening the mass of blood, to facilitate its circulation." The existence of plethora furnishes the indication for blood-letting, and the removal of this state constitutes the limit to which it may with propriety be carried. Carried beyond this limit, the detraction of blood can hardly fail to be pernicious." Injudiciously practised, blood-letting is injurious in proportion as it impoverishes the blood, and weakens the muscular power of the heart."

*Chronic Valvular Diseases of the Heart* are generally so far advanced before medical aid is called, that their cure is almost hopeless; but we can do much to retard their course. Their tendency is to produce hypertrophy and dilatation (as well as calcareous, cartilaginous, and other morbid deposits, in the valves), which greatly enhance their danger, and it is very desirable to obviate, or retard this effect. If, when this is the case, there is a plethoric condition of the system, with a flushed face, and full pulse, and the red globules of the blood in excess, moderate and occasionally repeated venesection, followed, if requisite, by local bleeding, will often give temporary relief, but should never be resorted to after the occurrence of dilitation. Some years ago I attended a lady with a valvular affection, of long standing, who, for months before her death, was unable to take the least exercise without producing severe dyspnœa; and the effort of walking across her room, at length induced her death. The condition of the heart, revealed by the *pose mortem* examination, caused surprise that life should have been so long maintained. There was a large calcareous deposit in the aortic and semilunar valves, converting them into a substance like stone, and to a less degree, in the mitral valve.

In the *Functional Affections of the Heart,* though bleeding is not so necessary as in the preceding classes of disease, yet it will be occasionally required.

*Palpitation,* depending on a variety of causes, must receive various treatment; but if caused by plethora, and where the pulse is full and strong, bleeding is beneficial; or if produced by spinal irritation, cups or leeches, applied to the tender portions of the spine, will be found to aid in relieving it.

In *Angina Pectoris,* during the paroxysm, if the system is plethoric, and the pulse strong, blood may be taken freely from the arm, but this is not often required. In connection with this, or alone, local bleeding may be resorted to. WALSHE remarks—"If the patient be the subject of undoubted sthenic plethora, and especially if the heart be known, by previous examinations, to be a well-nourished one, the abstraction of blood from a vein, or cupping between the shoulder blades, is clearly indicated; but bleeding must not be needlessly undertaken, and without assurance as positive as attainable, that the heart is, at least, not a dilated, soft, or flabby one." In all cases attended by debility, a pale skin, or anæmia, it would be injurious.

2D. THE BLOODVESSELS.

In *Asteritis,* where the usual symptoms of inflammation are present, free venesection, followed by leeching along the course of the inflamed vessel, will, in most cases be demanded.

In *Aneurisms,* where there is a rich condition of the blood, with a strong, full pulse, and a plethoric habit, and especially if there is inflammation of any of the thoracic or abdominal viscera, venesection may be practised moderately, with a view of lessening the volume and momentum of the blood, thus relieving the distension of the diseased vessel, and giving it an opportunity to contract. If the phlegmasiæ of the thorax or abdomen attend the aneurism, local bleeding is often necessary. Though free and repeated venesection was advocated by writers on the subject, fifty or more years ago, yet later, and prominent authors, as STOKES, WALSHE, FLINT, &c., confine themselves to one or two moderate bleedings in any case, and in the majority of cases, dispense with it altogether.

In *Arterial Palpitation*, depending upon congestion, or irritation of the spine, or solar plexus, accompanied with a full and excited pulse, venesection may be employed, though generally not necessary. Cups or leeches applied to the spine, or in case of palpitation of the abdominal aorta, leeches to the epigastrium are more frequently required.

In the early stages of *Phlebitis*, venesection is occasionally beneficial, especially where the pulse is full and frequent. Leeches along the course of the inflamed veins should be freely applied, and repeated should the inflammatory symptoms continue. In *crural Phlebitis*, or *Phlegmasia Dolens*, they should be applied along the course of the femoral vein. They may also be applied over *varicose veins*, where inflammation exists.

3d. In some of the diseases of the *Blood*, bleeding is strongly indicated; in others, it is wholly inapplicable.

In *Plethora*, where it is only temporary, it is not generally necessary to bleed; but if active, or threatening congestion of the brain or lungs, it should be freely employed.

*Purpura*, arising from opposite conditions of the system, requires different modes of treatment, depending on the cause of the disease. In some cases, as far as my observation goes, almost always, it arises from inefficient sanguification, and causes which debilitate. In cases of this nature, depletion would be highly improper; but it sometimes occurs in the robust and vigorous, and when this is the case, accompanied by a full, strong pulse, especially if there should be inflammatory congestion of the abdominal or thoracic viscera existing at the same time we may resort to venesection. It has been known to occur in suppressed menstruation, and in these cases, also, it would probably be beneficial to deplete.

Where the *Blood* and *Bloodvessels* are conjointly diseased, as in *hemorrhage*, it is frequently required. If, in hemorrhage, the pulse is full and strong, and there is fever; or if the patient is predisposed to plethora, venesection may be more or less freely practised. A large orifice should be made, so as to make a sudden impression, which will frequently check the hemorrhage. Where the hemorrhage is of a passive character, bleed ing is inadmissible. A few years ago, I was called in consultation to the case of a middle-aged lady, who had been bleeding freely for several hours from the nose, and over which all the remedies used appeared to have no control. She was also suffering with severe headache, and her pulse was moderately full and strong. With the assent of the physician attending her, I opened a vein in the arm, and bled her freely. A moment after we had examined her pulse, and found it still in good condition, she fainted, and it was a long time before she rallied; but the headache had disappeared, and there was no return of the *Epistaxis.*

In *Hæmoptysis,* the causes are so numerous and various, that no definite treatment can be recommended as applicable to all cases. When proceeding from pulmonary inflammations, from congestion or apoplexy of the lungs, from organic disease of the heart, from aneurism, from the suppression of habitual discharges (as the menses, &c.), from a morbid state of the blood, either in quality or quantity, from pregnancy, and occasionally from phthisis, bleeding is indicated, and may be repeated until the hemorrhage is checked, and the morbid excitement alleviated. When the pulse is full and strong, the system plethoric, and any of the above mentioned causes, except phthisis, producing it, we may use considerable latitude in resorting to the remedy; but if we have cause to suspect the existence of tubercles in the lungs, or a predisposition to them, we should be more guarded in our practice; and, if deemed necessary, use it moderately. If inflammatory irritation of the lungs should continue after venesection, or the subsidence of the hemorrhage, cups or leeches should be applied to the thorax. By lessening the quantity of blood by venesection, we relieve the bloodvessels of over-distension, moderate the force of the circulation, and modify the quality of the blood, by the reduction of its red corpuscles, rendering it less irritating or exciting.

In *Hæmatemesis,* arising from gastritis, or vascular irritation, where the pulse is active and strong, venesection may be required, but is seldom called for. If practised, the pulse should be the guide to the quantity taken. Where there is too much debility to admit of venesection, and inflammation or irritation are present, leeching the epigastrium may be substituted for general bleeding.

In *Melœna*, or intestinal hemorrhage, it is seldom necessary, but where this occurs in a plethoric habit of body, and an excited and strong pulse, it may be useful, either local or general. So in *Hemorrhoids*, accompanied by the same condition of the system as in melæna, it may also be resorted to. The local bleeding should be by leeches to the anus.

In *Hœmaturia*, where produced by local irritation, and not by remote causes, cups or leeches may be applied to the hypogastrium, loins, or perineum; or if not thus relieved, the lancet may be used.

In *Uterine Hemorrhage* of an active form, and especially if the system is plethoric, and the pulse full and strong, blood should be taken from the arm; and if symptoms of uterine congestion continue, local bleeding from surrounding parts may be necessary. But in the great majority of cases of uterine hemorrhage, the causes, and condition of the system, even in active hemorrhage, do not demand it; and in passive hemorrhage it should never be used.

I have thus given a brief view of the diseases of the organs of the circulation, and their contained fluid, in which bleeding is recommended by medical writers—though in my own practice I have judged it necessary in but a small minority of diseases of this character that have fallen to my care. There are but few diseases of this system in which depletion is not recommended, and from the function performed by it, and the immediate effect produced upon it, by the abstraction of blood, we might suppose the result would be prompt and decided upon them. Such is often, but not always the case; and if too freely or injudiciously resorted to, will frequently superinduce other diseases of more formidable characters than the one to be overcome. Such, for instance, is the case with phthisis, or anæmia. In constitutions predisposed to the former, the disease, which was latent, may be rendered active by the debility and impoverished state of the blood, produced by improper depletion; or the latter, by the impoverished condition of the blood, alone.

